# Do peer review models affect clinicians’ trust in journals? A survey of junior doctors

**DOI:** 10.1186/s41073-017-0029-8

**Published:** 2017-06-29

**Authors:** Jigisha Patel, Mary Pierce, Stephanie L. Boughton, Stephanie E. Baldeweg

**Affiliations:** 1 BioMed Central, part of Springer Nature, London, UK; 2 Retired. Independent researcher, London, UK; 3 University College London Hospitals NHS Foundation Trust and UCL Partners, London, UK

## Abstract

**Background:**

The aim of this survey was to determine the level of awareness and understanding of peer review and peer review models amongst junior hospital doctors and whether this influences clinical decision-making.

**Methods:**

A 30-question online anonymous survey was developed aimed at determining awareness of peer review models and the purpose of peer review, perceived trustworthiness of different peer review models and the role of peer review in clinical decision-making. It was sent to 800 trainee doctors in medical specialties on the University College London Partners trainee database.

**Results:**

The response rate was (178/800) 22%. Most respondents were specialist registrars. Checking that research is conducted correctly (152/178, 85%) and the data interpreted correctly (148/178, 83%) were viewed as the most important purposes of peer review. Most respondents were aware of open (133/178, 75%), double-blind (125/178, 70%) and single-blind peer review (121/178, 68%). 101/178 (57%) had heard of collaborative, 87/178 (49%) of post publication and 29/178 (16%) of decoupled peer review. Of those who were aware of double-blind, single-blind open and collaborative peer review, 85 (68%), 82 (68%), 74 (56%) and 24 (24%), respectively, understood how they worked. *The NEJM*, *Lancet* and *The BMJ* were deemed to have most trustworthy peer review, 137/178 (77%), 129/178 (72%) and 115/178 (65%), respectively. That peer review had taken place was important for a journal content to be used for clinical decision-making 152/178 (85%), but the ability to see peer review reports was not as important 22/178 (12%). Most felt there was a need for peer review training and that this should be at the specialist registrar stage of training.

**Conclusions:**

Junior hospital doctors view peer review to be important as a means of quality control, but do not value the ability to scrutinize peer review themselves. The unquestioning acceptance of peer review as final validation in the field of medicine emphasises not only the responsibility held by medical journals to ensure peer review is done well but also the need to raise awareness amongst the medical community of the limitations of the current peer review process.

**Electronic supplementary material:**

The online version of this article (doi:10.1186/s41073-017-0029-8) contains supplementary material, which is available to authorized users.

## Background

The underlying principle of peer review, the process of scrutiny of a piece of work by independent experts in the same field [1], is accepted as the best means by which research can be judged for publication [2]. Evidence-based medicine (EBM), where clinical practice is determined by the available evidence, is informed by peer reviewed literature [3]. Published literature influences clinical practice [4] and health policies [5], and so, it can be argued that the quality of peer review is important in determining the quality of evidence on which clinical practice is based.

While researchers view peer review as an essential part of scientific communication and a means of quality control [6], it has been recognized that there are flaws in the peer review system [7, 8]. Some of these criticisms, such as the potential for bias and needless delays, are being addressed. Opening [9] and blinding [10] peer review to address bias have been used and discussed for over a decade, and recent years have seen an increase in experimentation with different peer review models, including peer review *after* publication of the article [11] and independent peer review services that are separate from the journal [12, 13].

Although developed to provide fast and more efficient peer review, these innovations have yet to be evaluated for effectiveness and do not address the quality of peer review.

Furthermore, there is no objective evidence that peer review achieves the perceived aim of validating research [14]. The number of retractions of published articles is rising [15, 16], and it has been suggested that the majority of published research is not correct [17]. The open science movement [18], which advocates making research, peer review and raw data openly accessible to all not only furthers sharing and collaboration but also facilitates a process of self-correction as data can be validated by scrutiny and reproduction by others. However, for this to work, there needs to be recognition that there is a shift in responsibility towards the reader making a judgement based on all the ‘open’ information available to them including peer review, rather than unquestioningly accepting published literature that is peer reviewed as final validation. It is unclear how far this concept is embraced by the medical profession or how far the peer review process is considered to be relevant. This is an important question because in the UK, doctors are expected by their General Medical Council to be able to formulate research questions and apply findings from the literature to answer questions raised by specific clinical problems [19]. The method by which a piece of research was peer reviewed may become an important consideration for anyone who wishes to examine the published evidence to inform a clinical or policy decision.

To our knowledge, there is no research on doctors’ awareness or understanding of different peer review models or processes or whether peer review per se is a consideration when applying findings from the literature to answer specific clinical questions. We speculate that peer review is viewed as a standardized process that is the same from journal to journal and that there is little awareness or understanding amongst junior hospital doctors, defined as doctors at any stage of training between full registration with the General Medical Council and consultant level, of how different peer review models work.

The aims of this study were to determine:

- How aware junior doctors are of currently used peer review models.
- Whether different models of peer review influence junior doctors’ perception of trustworthiness of a published article.
- Whether a published article that has undergone peer review per se and knowledge of the model of peer review used influences clinical decision-making.
- Whether there is a perceived need for and desire to undergo peer review training.

## Methods

A 30-question survey was developed aimed at determining the demographic characteristics of the respondents, their academic activity, experience of peer review, views on the purpose of peer review, awareness of different peer review models and views about their trustworthiness and the role of peer review in clinical decision-making amongst the survey respondents [Additional file 1].

The survey was piloted by sending it to five test subjects who were not included in the final study. They provided feedback on the time to complete the survey, the clarity of the survey, how easy the questions were to understand and difficulties in completing the survey.

The survey was revised in response to their comments and created in electronic form using the commercially available service for creating online questionnaires, SurveyMonkey. It was sent by email to 800 trainee doctors in medical specialties on the University College London Partners trainee database. The email contained a link to the SurveyMonkey questionnaire which respondents completed anonymously. The researchers did not have access to information about who had completed the questionnaire. Two weeks later, a follow-up reminder email was sent to all trainees followed by three further emails each a week apart.

Some questions in the survey specifically referred to journals with:

- Closed peer review, where the peer reviewers remain entirely anonymous (*The Lancet, New England Journal of Medicine*)
- Open peer review, where the identity of the peer reviewers is known to the authors and peer review reports are published (*BMC Medicine*, *The BMJ*)
- Post publication peer review where peer review takes place after the article is published (*F1000Research*).

This group of journals was chosen because they publish medical research and represent a range of peer review models. Journals that operate collaborative peer review, where peer reviewers collaborate with each other and produce one joint report for authors to respond to, or decoupled review where peer review is conducted separately from the journal by a third party, were not selected because, as far as we were aware, there were no such journals that publish significant amounts of clinical research at the time of conducting this survey.

## Results

Two hundred and sixteen responses to the survey were received. Thirty-eight of these respondents provided demographic details about themselves, but did not respond to any questions about peer review. We present here the responses from the remaining 178. This gave a response rate of 22%. The demographic characteristics of the respondents are given in Table 1. The median age was 34 yrs (IQR 31 to 36 years). The majority of respondents were specialist registrars 152/178 (85%) and of those 85/152 (56%) were 5–10 years since qualification. 95/178 (53%) responded that they had no post graduate degree, while 61/178 (34%) had a Master’s degree, MD or PhD.

**Table 1:** Respondent’s characteristics

Gender	
Female	88
Male	88
Not answered	2
Time qualified	
0–5 years	35
5–10 years	89
>10 years	53
Not answered	1
Career stage	
Consultant	3
SPR	152
Core trainee	18
Other	3
Not answered	2
Post graduate degree	
None	95
Masters	24
MD	8
PhD	29
Other	20
Not answered	2

**Fig. 1:**
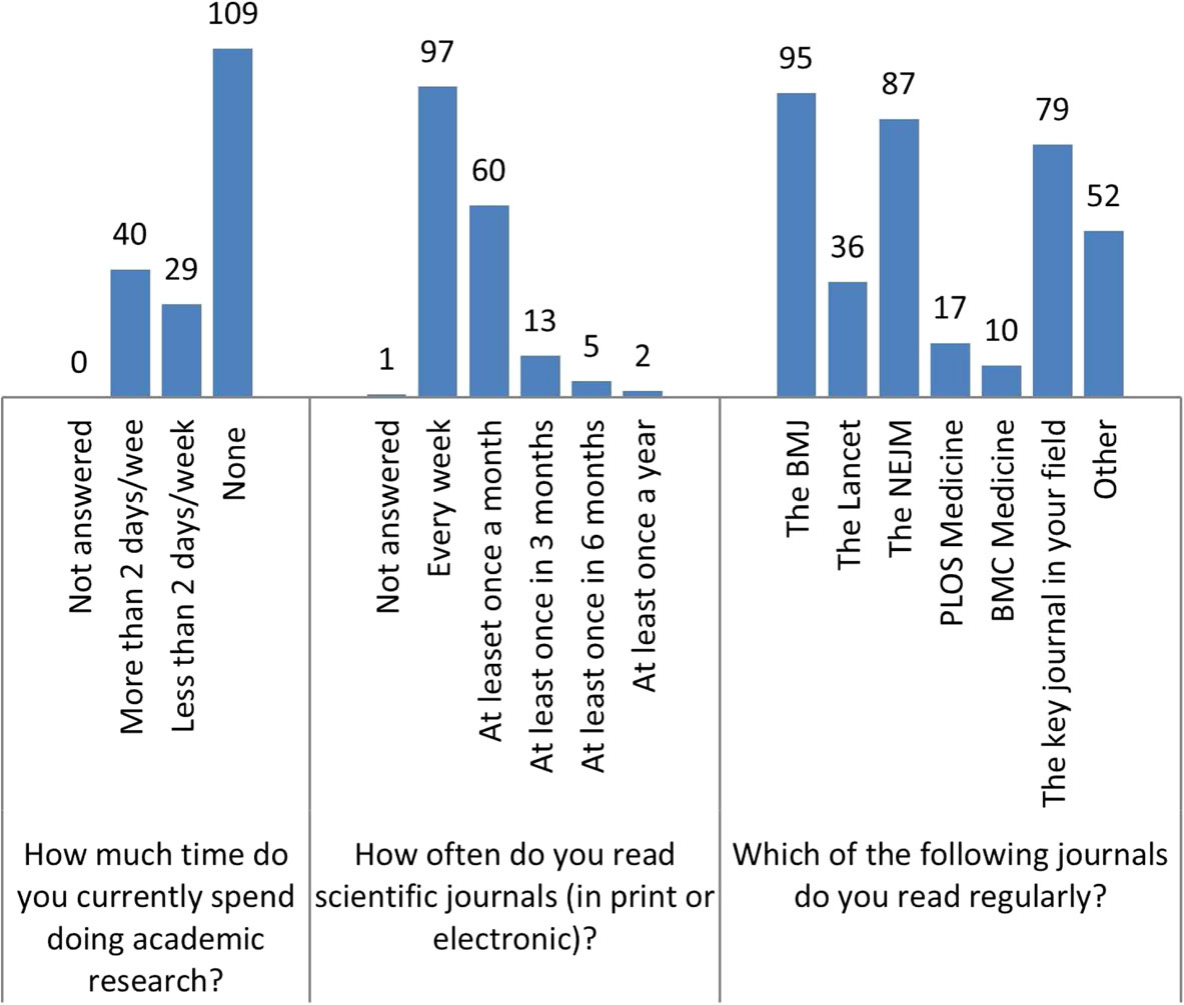
Academic activity

**Fig. 2:**
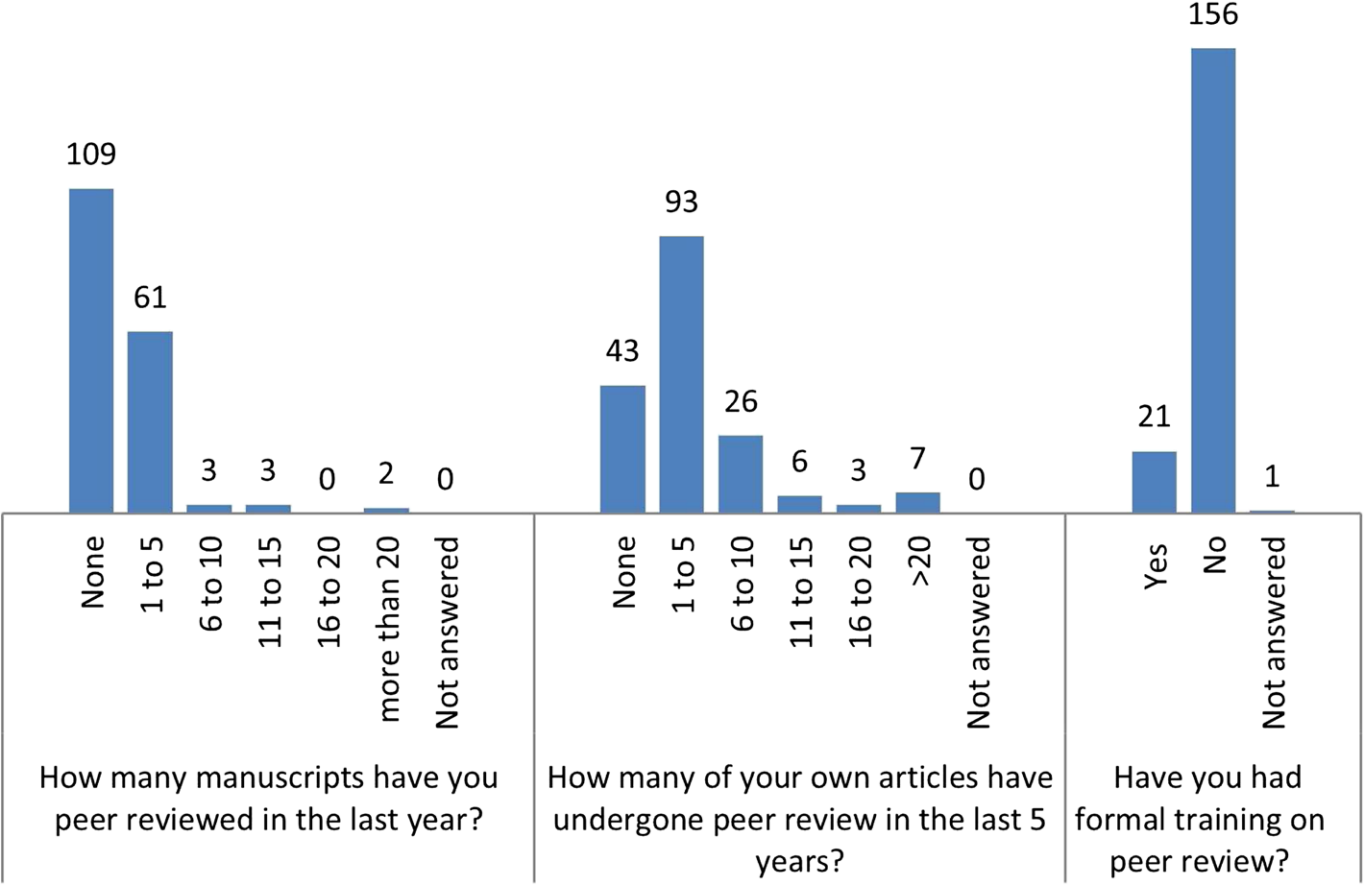
Experience of peer review

**Fig. 3:**
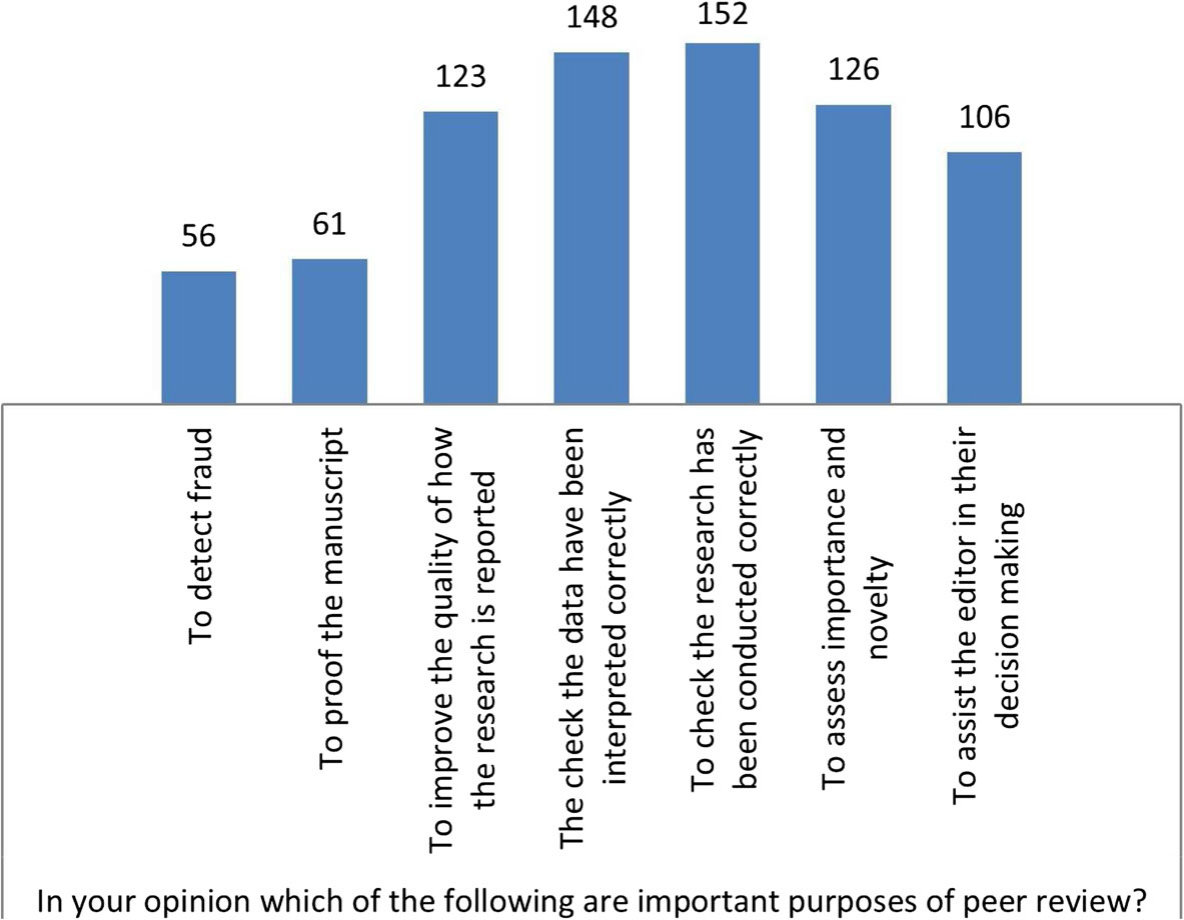
Purpose of peer review

**Fig. 4:**
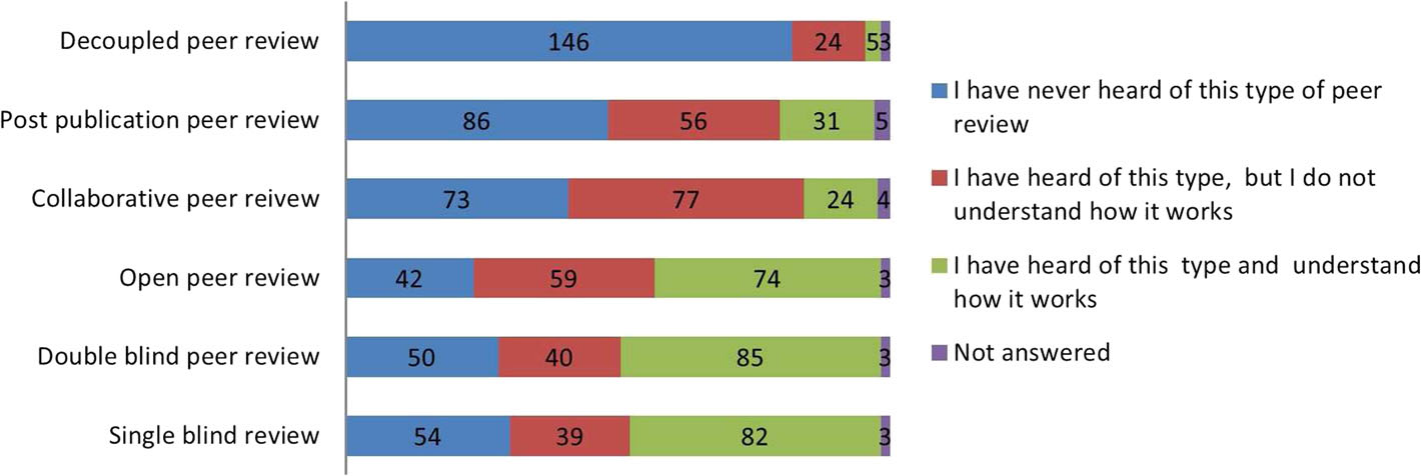
Awareness of innovations in peer review

**Fig. 5:**
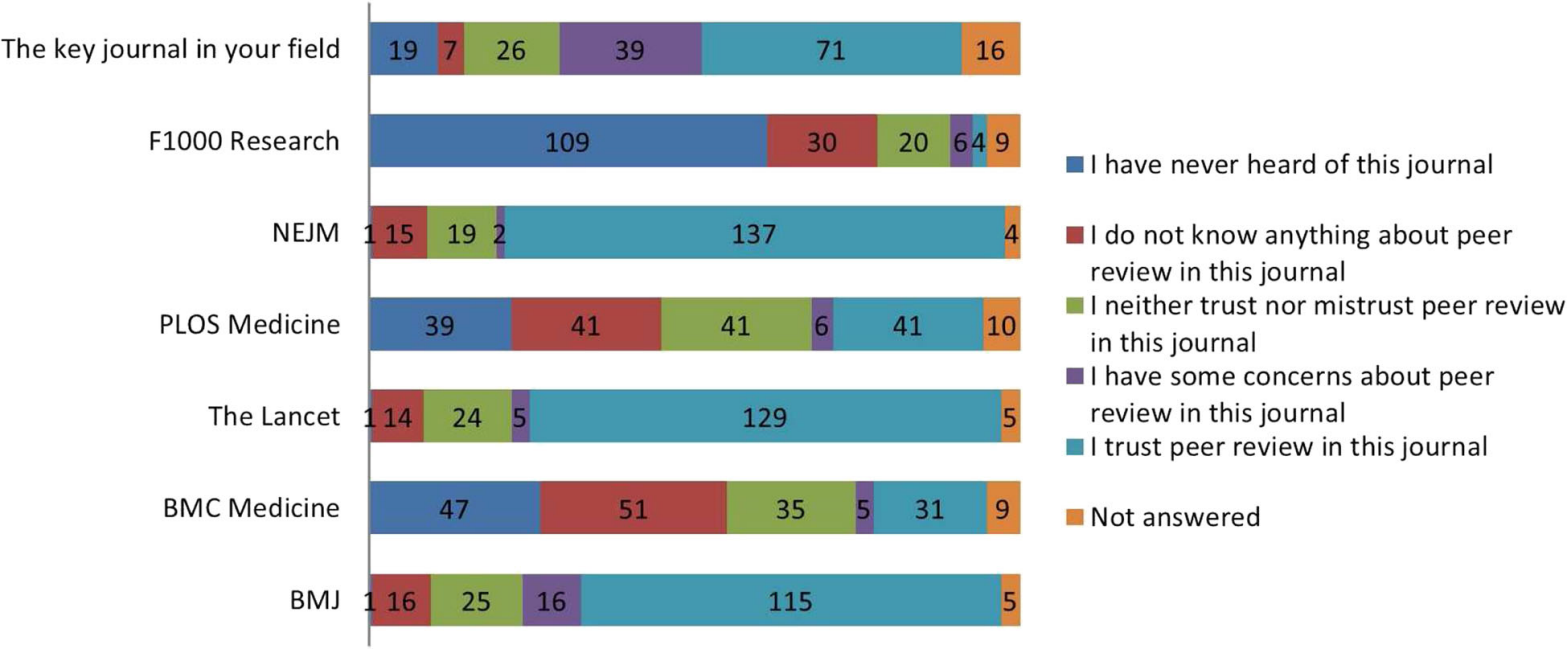
In response to ‘How far do you trust peer review in each of these journals?’

**Fig. 6:**
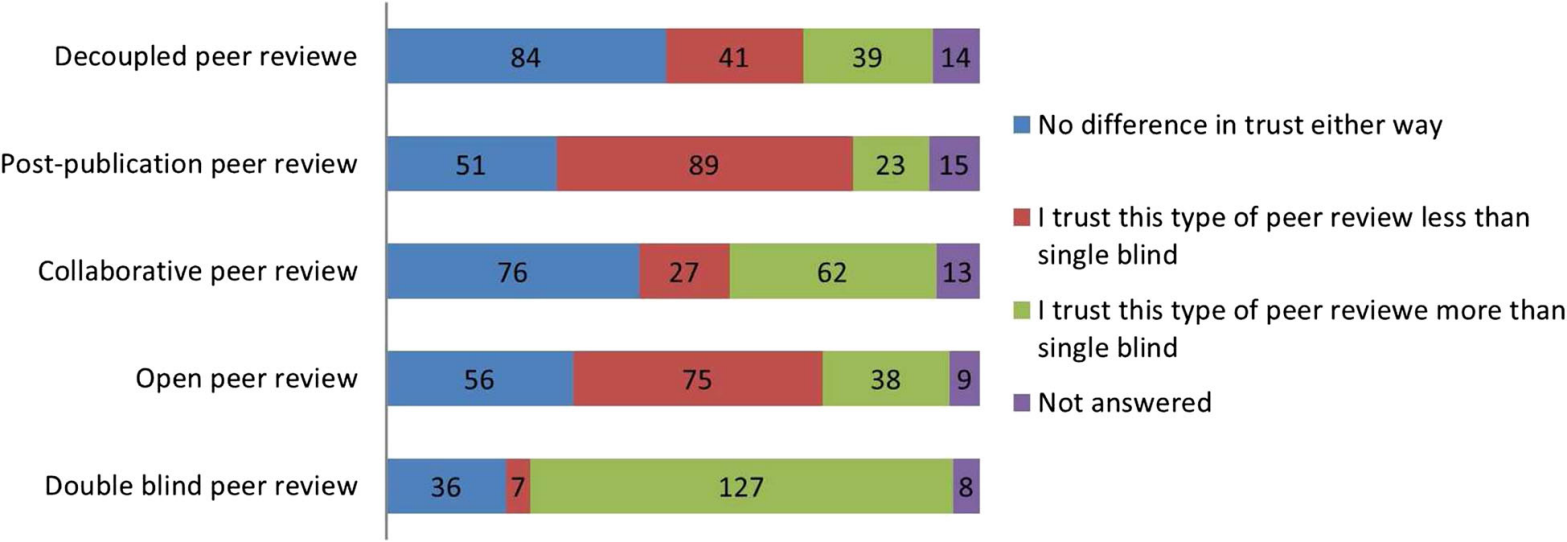
In response to ‘How far do you trust each type of peer review in comparison with single blind peer review?’

Responses to individual questions are shown in Figs. 1, 2, 3, 4, 5 and 6. The key findings are highlighted below.

### Academic activity

*The BMJ*, *New England Journal of Medicine* (*NEJM*) and the key journal in their field were the most frequent regularly read journals (Fig. 1).

### Experience of peer review

The majority of respondents 156/178 (88%) had not received formal training on peer review, although the majority had some experience of peer review; either peer review of their own work by others or peer reviewing others’ work themselves (Fig. 2).

### Purpose of peer review

Respondents were asked to select one or more important purposes of peer review. The most frequently selected purpose was ‘to check the research has been conducted correctly’ 152/178 (85%) followed by ‘to check the data have been interpreted correctly’ 148/178 (83%) (Fig. 3).

In a separate question, respondents were asked to rank each purpose of peer review as ‘Most important’, ‘Important’, ‘Somewhat important or ‘Not important’. To check that the research has been conducted correctly and to check the data have been interpreted correctly were ranked as most important by 93/178 (52%) and 81/178 (46%) respondents, respectively. ‘To improve the quality of how the research is reported’ was most important for 42/178 (24%) respondents. 34/178 (19%) ranked ‘to assess the importance and novelty of the research’ as the most important purpose of peer review, while 30/178 (17%) and 16/178 (9%) ranked ‘to detect fraud’ and ‘to help the editor in their decision-making’ as most important.

### Awareness of innovations in peer review

There was most awareness of open peer review, double-blind peer review and single-blind peer review, 133/178 (75%), 125/178 (70%) and 121/178 (68%), respectively. Of those who were aware of double-blind and single-blind peer review, a similar proportion understood how they worked, 85/125 (68%) and 82/121 (68%), respectively, while 74/133 (56%) of those who were aware of open peer review understood how it worked (Fig. 4).

### Peer review trustworthiness

*The NEJM* (single-blind peer review), *The Lancet* (single-blind peer review) and *The BMJ* (open peer review) received the most ‘I trust peer review in this journal’ responses, 137/178 (77%), 129/178 (72%) and 115/178 (65%), respectively, while *F1000 Research* received the most ‘I have never heard of this journal’ responses, 109/178 (61%). Interestingly, ‘the key journal in your field’ received the most ‘I have some concerns about peer review in this journal’, 39/178 (22%) [Additional file 2].

Respondents were then given a brief description of single-blind, double-blind, open, collaborative and decoupled peer review. They were asked how far they trusted each compared to single-blind peer review. Double-blind peer review was most frequently rated as more trustworthy than single-blind peer review (127/178, 71%) while post publication and open peer review were rated by more as ‘I trust this type of peer review less than single-blind peer review’, 89/178 (50%) and 75/178 (42%). Decoupled and collaborative peer review, each received a majority of ‘I would not change my trust in the article either way’ responses, 84/178 (47%) and 76/178 (43%), respectively, (Figs. 5 and 6).

### Peer review and clinical decision-making

When asked whether it was important to their clinical decision-making whether an article had been peer reviewed, 152/178 (85%) responded ‘yes’. When asked ‘When making a clinical decision, do you ever consider the type of peer review beyond whether an article has been peer reviewed?’, only 22/178 (12%) responded yes.

When asked ‘How often do you look up information in scientific journals to aid your clinical decision making?’, 74/178 (42%) responded that they do so at least once a week. When looking at an article to inform a clinical decision, 117/178 (66%) responded that they ‘Only consider articles published in journals where I trust peer review’. 44/178 (25%) responded that they never consider the type of peer review when making a clinical decision while only 3/178 (0.02%) responded that they only consider articles where they can see the peer reviewers’ reports.

### Peer review training

Respondents were asked whether they felt there was a need for peer review training during medical training and then, in a separate question, whether they would like to receive such training themselves. The majority (141/178, 79%) responded that they felt there was a need for peer review training. 24/178 (13%) did not feel there was a need for training, while 13/178 (0.1%) did not answer the questions. In response to whether they would like to receive training, the responses were similar: ‘yes’, 141/178 (79%); ‘no’, 24/178 (13%) and no answer, 13/178 (0.1%). However, 9 of those who responded that there was a need for peer review training responded that they would not like to receive such training themselves, while 9 different respondents who answered that there was no need for peer review training during medical training responded that they would like to receive peer review training themselves. When asked at what stage peer review training should be delivered, the responses were specialist registrar training 104, core medical training 80, medical school 77 and foundation years 48 (respondents were able to choose more than one option).

Additional file 3 shows the respondents’ free form comments at the end of the questionnaire. Nineteen respondents wrote further comments. Nine were in favour of training for peer review, 3 raised concerns about the added burden training would put on the medical curriculum and 3 commented on models of peer review.

Additional file 4 shows the distribution of responses according to the following subgroups: female, male, post graduate degree, no post graduate degree, experience of peer reviewing someone else’s work in the last year and no experience of peer reviewing someone else’s work in the last year. The distribution of responses to survey questions in these groups was similar to the distribution of responses for the 178 respondents as a whole. Additional file 5 shows the survey raw data.

### Limitations

This is a small observational study aimed at a specific group of junior doctors in training. The 22% response rate is very small. However, there have been response rates ranging from 2.7 to 10% in similar, albeit much larger, surveys on peer review [6, 20, 21].

There were three responses from consultants which suggest that although some doctors were in training when the database was compiled, by the time we sent the questionnaire out, they had been appointed to consultant posts or that the survey was forwarded beyond the original cohort of 800 trainee junior doctors for whom it was intended. Because of the decision to distribute the survey using an online method, it was not possible to collect data on all potential responders who had the opportunity to complete the questionnaire and to compare the responders with the non-responders to this survey. Had we been able to identify the non-responders, we might have been able to use different approaches to increase the response rate, by for example, sending them personalized electronic or paper questionnaires or by telephoning to ask them to complete the questionnaire.

Due to the high level of competition for jobs in London and a national selection process that credits research and other academic activities as well as clinical skills, it might be expected that our sample included a greater proportion with experience of scientific research and other academic activities than a country wide sample of junior doctors and may not be a representative sample of junior hospital doctors in the UK.

This survey did not aim to further explore the responses provided by the participants, for example, it did not seek explanations from participants the reasons for their preference or otherwise of certain journals or models of peer review.

There is no comparative analysis because the subgroups were too small to obtain meaningful results in a comparative analysis.

Given the small size of this survey, low response rate and selected group of the respondents, it would be unwise to generalize our findings to the wider population of junior hospital doctors in the UK.

## Discussion

To the best of our knowledge, this is the first survey of awareness of peer review models and its influence on clinical decision-making amongst a group of junior doctors in training. It is the first survey to specifically explore understanding and perceived trustworthiness of different models of peer review in this group.

The most notable finding from this survey is that respondents trust familiar established names. *The BMJ, NEJM* and *the Lancet* were viewed to have trustworthy peer review by the majority of respondents. More respondents stated that they trusted peer review in these journals than stated that they understood the type of peer review conducted by these journals. There was little awareness of the type of peer review in specific journals that might allow doctors to critically appraise the peer review process themselves.

The two most highly rated purposes of peer review in the opinion of our cohort were to check that research is conducted correctly and the data interpreted correctly. These suggest that the majority of respondents appear to view peer review as a process of validation. Previous surveys have reported the view that peer review is the only means of control of scientific information [6] and have ranked improving the quality of a manuscript as an important purpose of peer review [6, 20, 21].

Once provided with an explanation of the models of peer review, double-blind peer review was deemed by most to be more trustworthy than single-blind. Double-blind peer review has been noted to be preferred by researchers [6, 20]. However, the latest report by the Peer Review Consortium found that this opinion is changing with researchers viewing double-blind as equal to single- and open peer review [21].

Paradoxically, even after receiving an explanation of each model, those peer review models that offer more opportunity for scrutiny, open and post publication peer review, were deemed by most to be less trustworthy than single-blind peer review. Only three responded that they only trust research published in journals where they can see the peer review reports. This suggests that this group of junior hospital doctors do not view the ability to scrutinize peer review themselves as important. What seems to be important is that it is scrutinized by someone else they deem to be trustworthy, such as the journal editor or group of peer reviewers. Openness per se does not seem to be valued. This is interesting because open peer review was pioneered in the field of clinical medicine for ethical reasons, the argument being that all involved in research that affects patient care should be accountable for their decisions and open to scrutiny [22].

That research is peer reviewed is valued as important to clinical decision-making, but the type of peer review appears to be of much less importance. Half of respondents were not involved in academic research, peer review or weekly reading of academic journals. This study did not explore which sources of information junior doctors use for clinical decision-making, and it is possible that doctors do not view the published evidence as important in their day to day work because much of recommended best clinical practice is encompassed in resources such as the National Institute for Health and Care Excellence (NICE) guidelines [23], guidance by the Royal Colleges such as the Royal College of Physicians [24], Cochrane systematic reviews [4] and specialty specific societies. In addition, hospitals often have their own clinical guidelines and there are senior colleagues available to give advice. Junior hospital doctors may feel they do not need to directly refer to primary research in their day to day work or to make independent decisions with all of these resources available to them.

The majority of respondents wanted training on how to peer review themselves and thought peer review training should be part of medical training. We speculate that doctors recognize the importance of peer review and may view it as an important skill to develop in their medical career when focusing on academic work, but consider it less important when making clinical decisions.

## Conclusions

This is the first study to specifically explore the views of a group of junior hospital doctors on peer review, peer review models and their influence on trust in journals. This group of doctors had little awareness of newer peer review models and appeared to put their trust in familiar journal names with little desire to scrutinize peer review themselves. It is not surprising that there is an expectation that peer review, regardless of the model used, will be done correctly and will not need further scrutiny. This faith in peer review may be misplaced in light of the concept of open science which, by encouraging openness and sharing of the scientific process and data, acknowledges that there is uncertainty in and a continuing need to verify published findings, even when they have been peer reviewed. The unquestioning acceptance of peer review as final validation in the field of medicine emphasises not only the responsibility held by medical journals to ensure peer review is done well but also the need to raise awareness amongst the medical community of the limitations of the current peer review process.

## Additional files

Additional file 1: The questionnaire. (PDF 814 kb)

Additional file 2: Responses to ‘the key journal in your field’. (DOCX 17 kb)

Additional file 3: Responses to ‘Do you have any further comments or suggestions’. (DOCX 19 kb)

Additional file 4: Survey responses according to gender, post graduate degree and recent experience of peer review. (PDF 437 kb)

Additional file 5: Survey raw data. (XLSX 68 kb)
