# Supplementary material for: Do peer review models affect clinicians’ trust in journals? A survey of junior doctors
Source: Res Integr Peer Rev. 2017 Jun 29;2:11. doi: 10.1186/s41073-017-0029-8 (PMC5803626; doi:10.1186/s41073-017-0029-8)
Supplement: Supplementary file 1 — The questionnaire. (PDF 814 kb) [file 41073_2017_29_MOESM1_ESM.pdf]

–

3%

Thank you for taking the time to fill out our brief survey. The survey takes approximately 10 minutes to complete and your answers will be kept anonymous.

The purpose of this study is to establish a peer review process specifically for medicine and to evaluate the need for training on peer review. We hope that the results of this study will inform the development of training in peer review.

Please do not look anything up while completing the survey. Please answer the questions sequentially and do not revisit previous questions in the survey.

We very much appreciate your participation!

Please click “Next” to start the survey.

Next

–

6%

Age in years
(Enter number)

Prev Next

–

9%

Gender

- ◯ Male
- ◯ Female

Prev Next

–

13%

At what stage are you in your career?

- ◯ Core Medical Trainee
- ◯ Specialist Registrar
- ◯ Consultant
- ◯ Other (e.g. Clinical Assistant, Associate Specialist) please specify

Prev Next

Springer BioMed Central The Open Access Publisher UCLPartners Academic Health Science Partnership

–

16%

How long have you been qualified (full GMC registration)?

- 0 years up to/ including 5 years
- More than 5 years up to/including 10 years
- More than 10 years up to/including 15 years
- More than 15 years up to/including 20 years
- More than 20 years

Prev Next

Springer BioMed Central The Open Access Publisher UCLPartners Academic Health Science Partnership

–

19%

What is your area of specialisation?

Prev Next

–

22%

Do you have a post-graduate degree? (if so, please select your highest degree)

- No
- Masters
- MD
- PhD
- Other (please specify)

Prev Next

–

25%

How much time do you currently spend doing academic research?

- None
- Less than 2 days a week
- 2 days or more a week

Prev Next

–

28%

How often do you read scientific journals (in print or electronic)?

- ◯ Every week
- ◯ At least once a month
- ◯ At least once every 3 months
- ◯ At least once every 6 months
- ◯ At least once a year
- ◯ Less than once a year

Prev Next

–

31%

Which of the following journals do you read regularly? (Select all that you read regularly)

- ☐ The BMJ
- ☐ The Lancet
- ☐ The New England Journal of Medicine
- ☐ PLOS Medicine
- ☐ BMC Medicine
- ☐ The key journal in your field*
- ☐ Other (please specify)

____

*** The key journal in your field, please specify**

____

Prev Next

–

34%

Have you had <u>formal</u> training on peer review?

- ◯ Yes
- ◯ No

Prev Next

–

38%

How many of your own articles have undergone peer review in the past 5 years?

- ◯ 0
- ◯ 1 to 5
- ◯ 6 to 10
- ◯ 11 to 15
- ◯ 16 to 20
- ◯ more than 20

Prev Next

Springer BioMed Central The Open Access Publisher UCLPartners Academic Health Science Partnership

–

44%

In your opinion, which of the following are important purposes of peer review? (please select as many as you feel are relevant)

- [ ] To assist the editor in their decision making
- [ ] To assess importance and novelty
- [ ] To check the research has been conducted correctly
- [ ] The check the data have been interpreted correctly
- [ ] To improve the quality of how the research is reported
- [ ] To proof the manuscript
- [ ] To detect fraud

Prev Next

47%

Please assess the importance of the following for peer review.

	Not important	Somewhat important	Important	Most important
To assist the editor in their decision making	○	○	○	○
To assess importance and novelty	○	○	○	○
To check the research has been conducted correctly	○	○	○	○
The check the data have been interpreted correctly	○	○	○	○
To improve the quality of how the research is reported	○	○	○	○
To proof the manuscript	○	○	○	○
To detect fraud	○	○	○	○

Prev Next

Springer BioMed Central The Open Access Publisher UCLPartners Academic Health Science Partnership

–

50%

How aware are you of the following types of peer review?

	I have never heard of this type<br<font size="-2">	I have heard of but don't know how this type works	I have heard of and understand how this type works
Single blind peer review	○	○	○
Double blind peer review	○	○	○
Open peer review	○	○	○
Collaborative peer review	○	○	○
Post publication peer review	○	○	○
Decoupled peer review	○	○	○

Springer BioMed Central The Open Access Publisher UCLPartners Academic Health Science Partnership

–

53%

How often do you look up information in scientific journals to aid your clinical decision making?

- ○ At least once a week
- ○ At least once a month
- ○ At least once every 3 months
- ○ At least once every 6 months
- ○ At least once a year
- ○ Less than once a year

Prev Next

Springer BioMed Central The Open Access Publisher UCLPartners Academic Health Science Partnership

–

56%

Is it important to your decision making whether an article has been peer reviewed?

- Yes
- No

Prev Next

Springer BioMed Central The Open Access Publisher UCLPartners Academic Health Science Partnership

–

59%

When making a clinical decision, do you ever consider the type of peer review in your decision making beyond whether an article has been peer reviewed?

- Yes
- No

Prev Next

Please rate the following journals for trustworthiness of peer review:

	I have never heard of this journal	I do not know anything about peer review in this journal	I have some concerns about peer review in this journal	I do not trust or mistrust peer review in this journal	I trust peer review in this journal
The BMJ	○	○	○	○	○
BMC Medicine	○	○	○	○	○
The Lancet	○	○	○	○	○
PLOS Medicine	○	○	○	○	○
The New England Journal of Medicine	○	○	○	○	○
F1000 Research	○	○	○	○	○
The key journal in your field	○	○	○	○	○

Prev Next

Springer BioMed Central The Open Access Publisher UCLPartners Academic Health Science Partnership

–

66%

Here is a brief description of some of the types of peer review previously mentioned.

- **Single blind peer review:** Reviewers are aware of the authors' identity but reviewers remain anonymous.
- **Double blind peer review:** Reviewers are blinded to the authors' identities and authors do not know the identity of the reviewers.

Single blind peer review is the most frequently used type of peer review and the one most researchers have experienced. How far would you trust double blind peer review in comparison to single blind peer review?

○ I would trust double blind peer review more than single blind peer review

○ I would not change my trust in the article either way

○ I would trust double blind peer review less than single blind peer review

Prev Next

Springer BioMed Central The Open Access Publisher UCLPartners Academic Health Science Partnership

–

69%

Here is a brief description of some of the types of peer review previously mentioned.

- **Single blind peer review:** Reviewers are aware of the authors' identity but reviewers remain anonymous.
- **Open peer review:** Peer review is not anonymous. Reviewers and authors are aware of each other's' identities. If the article is published, the reviewers signed reports may also be posted online alongside the published article.

Single blind peer review is the most frequently used type of peer review and the one most researchers have experienced. How far would you trust open peer review in comparison to single blind peer review?

- ○ I would trust open peer review more than single blind peer review
- ○ I would not change my trust in the article either way
- ○ I would trust open peer review less than single blind peer review

Prev Next

Springer BioMed Central The Open Access Publisher UCLPartners Academic Health Science Partnership

–

72%

Here is a brief description of some of the types of peer review previously mentioned.

- **Single blind peer review:** Reviewers are aware of the authors' identity but reviewers remain anonymous.
- **Collaborative peer review:** Reviewers discuss the article among themselves and agree one set of revisions and produce one report so that a unified decision can be presented to the authors.

Single blind peer review is the most frequently used type of peer review and the one most researchers have experienced. How far would you trust collaborative peer review in comparison to single blind peer review?

- ○ I would trust collaborative peer review more than single blind peer review
- ○ I would not change my trust in the article either way
- ○ I would trust collaborative peer review less than single blind peer review

Prev Next

–

75%

Here is a brief description of some of the types of peer review previously mentioned.

• **Single blind peer review:** Reviewers are aware of the authors' identity but reviewers remain anonymous.
• **Post publication peer review:** Articles are posted online after brief editorial checks. Peer review is conducted after publication. Articles are marked to indicate whether peer review has taken place yet.

Single blind peer review is the most frequently used type of peer review and the one most researchers have experienced. How far would you trust post publication peer review in comparison to single blind peer review?

- ◯ I would trust post publication peer review more than single blind peer review
- ◯ I would not change my trust in the article either way
- ◯ I would trust post publication peer review less than single blind peer review

Prev Next

–

78%

Here is a brief description of some of the types of peer review previously mentioned.

• **Single blind peer review:** Reviewers are aware of the authors' identity but reviewers remain anonymous.
• **Decoupled peer review:** Peer review is conducted by a service external to the journal and peer reviewer reports submitted to the editor alongside the article.

Single blind peer review is the most frequently used type of peer review and the one most researchers have experienced. How far would you trust decoupled peer review in comparison to single blind peer review?

- ◯ I would trust decoupled peer review more than single blind peer review
- ◯ I would not change my trust in the article either way
- ◯ I would trust decoupled peer review less than single blind peer review

Prev Next

–

81%

When looking at an article to inform a clinical decision,

- I never consider peer review of the article
- I only read articles published in journals where I trust the peer review process
- I only trust articles where I can see the peer review reports myself

Prev Next

–

81%

When looking at an article to inform a clinical decision,

- I never consider peer review of the article
- I only read articles published in journals where I trust the peer review process
- I only trust articles where I can see the peer review reports myself

Prev Next

–

84%

Do you feel that there is a need for training in peer review during medical training?

- ◯ Yes
- ◯ No

Prev Next

–

88%

If yes, please indicate at which stage of medical training peer review training should be required (you may select more than one answer):

- ☐ Medical School
- ☐ Foundation training
- ☐ Core medical training
- ☐ Higher specialist training

Prev Next

Springer BioMed Central The Open Access Publisher UCLPartners Academic Health Science Partnership

_

91%

Would you like to receive peer review training?

- Yes
- No

Prev Next

Springer BioMed Central The Open Access Publisher UCLPartners Academic Health Science Partnership

_

94%

If yes, would you prefer training to be:

- Online
- Face to face

Prev Next

Springer BioMed Central The Open Access Publisher UCLPartners Academic Health Science Partnership

–

97%

Do you have any further comments or suggestions?

Prev Next

Springer BioMed Central The Open Access Publisher UCLPartners Academic Health Science Partnership

–

100%

Thank you for taking the time to complete this survey.

Prev Done
