# Supplementary material for: Do peer review models affect clinicians’ trust in journals? A survey of junior doctors
Source: Res Integr Peer Rev. 2017 Jun 29;2:11. doi: 10.1186/s41073-017-0029-8 (PMC5803626; doi:10.1186/s41073-017-0029-8)
Supplement: Supplementary file 2 — Responses to ‘the key journal in your field’. (DOCX 17 kb) [file 41073_2017_29_MOESM2_ESM.docx]

|  |
| --- |
|  |
| BGS |
| JCEM, Bone, Thyroid |
| GUT |
| PN, JNNP |
| Age & Ageing |
| Thorax |
| Clinical Infectious Diseases |
| Journal of Infection |
| JCEM |
| jcem |
| Lancet ID/CID/PLOS NTDs |
| Thorax |
| journal antimicrobial chemotherapy |
| malaria journal |
| CID, Lancet ID, Journal of Infection |
| Journal of anti microbial chemotherapy |
| HIV Medicine |
| Diabetic Medicine, Diabetes Care, JCEM, Clin Endo, Nature reviews Endocrinology |
| HIV Medicine, |
| Palliative Medicine |
| Thorax |
| Gut |
| GUT |
| American journal of respiratory and critical care medicine |
| EJCVI |
| Rheumatology |
| EHJ, JACC, Heart, Circulation |
| thorax |
| Nature |
| Gut. |
| IJTLD |
| Circulation |
| Gut |
| NDT, KI, CKJ, CJASN, Age and Ageing |
| Journal of the American Society of Nephrology |
| Thorax |
| European journal of endocrinology |
| AJRCCM |
| JCEM |
| Thorax |
| BRAIN, NEUROLOGY, ANNALS OF NEUROLOGY |
| JCEM |
| Clinical Endocrinology |
| Endocrinology |
| Thorax |
| Thorax, ERJ |
| AJRCCM |
| Thorax |
| Gut |
| British Journal of Sports Medicine |
| Blue Journal |
| Thorax |
| STD and aids |
| Infection, HIV Medicine |
| Thorax |
| BJD |
| CJASN, KI, NDT, CKJ |
| BJD |
| Neurology |
| Palliative Medicine |
| JCEM |
| age and ageing |
| British journal of dermatology |
| JASN |
| Lancet ID |
| Neurology, practical neurology, JNNP |
| JICM |
| Journal of palliative medicine |
| Hearing, Balance and Communication |
| Circulation |
| Thorax |
| Neurology |
| American Journal of Respiratory and Critical Care Medicine, Thorax |
| Circulation |
| CJASN |
| AJRCCM, Critical Care Medicine, Intensive Care Medicine, Critical Care |
| JACC, Circulation |
| Brain |
| Circulation, JACC, JAHA |
| AIDS |
| Gut gastro ibd jcc |
| Gastroenterology |
| Circulation |
| Hepatology |
| Age and ageing |
| Multiple |
|  |
| CID, EID, JI |
| Brain |
