# Supplementary material for: Do peer review models affect clinicians’ trust in journals? A survey of junior doctors
Source: Res Integr Peer Rev. 2017 Jun 29;2:11. doi: 10.1186/s41073-017-0029-8 (PMC5803626; doi:10.1186/s41073-017-0029-8)
Supplement: Supplementary file 3 — Responses to ‘Do you have any further comments or suggestions’. (DOCX 19 kb) [file 41073_2017_29_MOESM3_ESM.docx]

- I never really knew much about this, but having completed the survey, I don't feel like I am missing out on much.
- interesting survey. It would be good to hear the results and any action proposed from it.
- I peer review already so do not need training but it would be useful for those not experienced.
- Need for critical appraisal and research methodology training in general.
- Peer review makes reader think they are reading good science but in reality it is not a very good process at all. However there is nothing else better out there at this moment in time
- I received training in the US during my PhD and by asking supervisors. It would be great to introduce this early in the UK along with training on grantwriting and publishing. We also need to implement mentorship (which is assessed for quality).
- Probably could have been face-to-face during earlier stages of training eg in weekly foundation or cmt training-as spr online would be best
- My background and career aims are academic. I have had multiple periods of training in appraisal of papers and believe critical appraisal of peer review is central to interpretation of papers and should be included at all levels of training (as should regular tutor facilitated application of primary research to clinical questions at all levels of clinical practice).
- Double-blind peer review improves the situation whereby publications are suppressed by friction in some fields. However, in some small fields, where all of the reviewers and authors are aware of each other and their work, it cannot be truly double-blind unless a reviewer from another field is also involved. We get to see a lot of research which is published as a result of a reviewer/author circuit, but we do not see other research, which may have more scientific merit which has not been published due to one or two dominant figures within some fields. This effect can end up leading whole fields astray - with the bulk of published research and investment in research being directed towards the interests of certain members and not with clinical priority. The importance of what is not published is not discussed - this is the benefit of post-publication review. I have worked in such a field, and the extraordinary lack of transparency and manipulation of the publication process should be better regulated by a GMC style body. Until this happens, it will be difficult to encourage clinicians to stay in science.
- Any additional input into training should not consist of any more "workplace based assessments" as there are too many of these at the moment. Thank you.
- Peer review training will be an important addition to the training curriculum. I feel this should definitely be a part of the training and hope I get to attend one before I complete my training.
- Medical schools should be teaching peer review as part of critical appraisal.
- This should be considered as part of overall training in Evidence Based practice as part of understanding the limitations to it's interpretation alongside other factors such as publication bias etc.
- Peer Review training would be a great idea!
- When looking at an article to inform a clinical decision,: None of the answers to this question applied as it did not cover enough eventualities eg "I read non-peer reviews articles but have a more critical approach" I was trained in Peer review at Medical School - we did a critical thinking module in final year which was fantastic (Liverpool 2001). We had to write a literature review and then critically appraise other people's literature reviews. I think there were about four steps to the process e.g. changing our review as per reviewer's comments etc. It was really educational and that model could be used for trained Drs too.
- A plea: please UCLP focus on getting the providers to deliver on the basics of training before we use resource on adding value in other ways.
- For the small number of registrars who go onto become consultants with a strong interest in academia a thorough understanding the peer review process is of course crucial. However, introducing a compulsory module for all registars will be a waste of time for many, and time is the one thing we are the most short of. Please consider carefully the implications of adding yet another strand to our already bloated curricula before embarking on this process.
- Peer review is never anonymous. You can usually work out who it is. Therefore I favour transprency and possibly publication of peer review comments
- The main issue in peer review is the biases of the reviewers. The Frontiers model, where reviewers' names appear on the manuscript is a good idea.

Summary

19 comments altogether

9 comments in favour of peer review training.

3 comments that raised concerns about the added burden peer review training would add to the medical curriculum.

3 were comments on different peer review models (collaborative, open and double blind peer review)
