# Supplementary material for: Do peer review models affect clinicians’ trust in journals? A survey of junior doctors
Source: Res Integr Peer Rev. 2017 Jun 29;2:11. doi: 10.1186/s41073-017-0029-8 (PMC5803626; doi:10.1186/s41073-017-0029-8)
Supplement: Supplementary file 4 — Survey responses according to gender, post graduate degree and recent experience of peer review. (PDF 437 kb) [file 41073_2017_29_MOESM4_ESM.pdf]

Additional file 4

Survey responses according to gender, post-graduate degree and recent experience of peer review. (%)						
	GENDER		Post-graduate degree (PGD)		Peer reviewed (PR) a manuscript in the last year	
	Female (n=88)	Male (n=88)	No PGD (n=95)	PGD (n=81)	No PR last year(n=109)	Some PR last year (n=69)
**Gender**						
Female	88		45(47)	43(53)	56(51)	37(54)
Male		88	50(53)	38(47)	51(47)	32(46)
not answered	_	_	0	1(1)	2(2)	0
**How long have you been qualified (Full GMC registration)?**						
0-5yrs	17 (19)	17 (19)	28(29)	7(9)	31(28)	4(6)
5-10yrs	44 (50)	45 (51)	52(55)	37(46)	54(50)	35(51)
>10yrs	27 (31)	26(30)	15(16)	38(47)	23(21)	30(43)
Not answered	0	0	0	0	1(1)	0
**At what stage are you in your career?**						
Consultant	2 (2)	1(1)	2(2)	1(1)	2(2)	1(1)
SPR	78 (89)	73(83)	76(80)	76(94)	88(81)	64(93)
Core trainee	6 (7)	12(14)	15(16)	3(4)	15(14)	3(4)
Other	1 (1)	2(2)	2(2)	1(1)	2(2)	1(1)
Not answered	1(1)	0	0	1(1)	2(2)	0
**Do you have a post-graduate degree (select the highest)?**						
None	45 (51)	50(57)	95(100)	0	69(63)	26(38)
Masters	11 (13)	12(14)		24(30)	11(10)	13(19)
MD	5 (6)	3(3)		8(10)	3(3)	5(7)
PhD	13(15)	16(18)		29(36)	10(9)	19(28)
other	13 (15)	7(8)		20(25)	14(13)	6(9)
Not answered	1(1)	0		_	2(2)	0
**How much time do you currently spend doing academic research?**						
None	60 (68)	47(53)	65(68)	44(54)	83(76)	26(38)
Less than 2 days/week	9 (10)	20(23)	15(16)	14(17)	18(17)	11(16)
More than 2 days/wee	19 (22)	21(24)	15(16)	25(31)	8(7)	32(46)
Not answered	0	0	0	0	0	0
**How often do you read scientific journals (in print or electronic)?**						
At least once a year	2 (2)	0	2(2)	0	2(2)	0
At least once in 6 months	2 (2)	3(3)	4(4)	1(1)	4(4)	1(1)
At least once in 3 months	10 (11)	3(3)	8(8)	5(6)	12(11)	1(1)
At leaset once a month	31 (35)	28(32)	40(42)	20(25)	43(39)	17(25)
Every week	43 (49)	54(61)	41(43)	56(69)	47(43)	50(72)
Not answered	0	0	0	0	1(1)	0
**which of the following do you read regulary?**						
The BMJ	47	48	57(60)	38(47)	67(61)	28(41)
The Lancet	14	22	13(14)	23(28)	14(13)	22(32)
The NEJM	38	49	36(38)	51(63)	42(39)	45(65)
PLOS Medicine	9	8	3(3)	14(17)	9(8)	8(12)
BMC Medicine	4	6	2(2)	8(10)	5(5)	5(7)
The key journal in your field	39	39	39(41)	44(54)	42(39)	37(54)
Other	28	24	29(31)	29(36)	34(31)	18(26)
**Have you had formal training on peer review?**						
Yes	9 (10)	12(14)	13(14)	8(10)	12(11)	9(13)
No	78 (89)	76(86)	82(86)	74(91)	96(88)	60(87)
Not answered	1 (1)	0	0	1(1)	1(1)	0
**How many of your own articles have undergone peer review in the last 5 years?**						
None	26 (30)	16(18)	27(28)	16(20)	40(37)	3(4)
1 to 5	45 (51)	47(53)	52(55)	41(51)	64(59)	29(42)
6 to 10	12 (14)	14(16)	11(12)	15(19)	4(4)	22(32)
11 to 15	2 (2)	4(5)	2(2)	4(5)	0	6(9)
16 to 20	2 (2)	1(1)	0	3(4)	1(1)	2(3)
>20	1 (1)	6(7)	3(3)	4(5)	0	7(10)
Not answered	0	0	0	0	0	0
**How many manuscripts have you peer reviewed in the last year?**						
None	56 (64)	51(58)	69(73)	40(49)	109	0
1 to 5	30 (34)	31(35)	24(25)	37(46)	0	61(88)
6 to 10	1 (1)	2 (2)	1(1)	2(2)	0	3(4)
11 to 15	1 (1)	2 (2)	0	3(4)	0	3(4)
16 to 20	0	0	0	0	0	0
more than 20	0	2 (2)	1(1)	1(1)	0	2(3)
Not answered	0	0	0	0	0	0

**In your opinion which of the following are important purposes of peer review?**						
To assist the editor in their decision making	48(55)	58(66)	49(52)	57(70)	57(52)	49(71)
To assess importance and novelty	66(75)	59(67)	65(68)	61(75)	71(65)	55(80)
To check the research has been conducted correctly	73(83)	79(90)	83(87)	69(85)	91(83)	61(88)
The check the data have been interpreted correctly	72(82)	75(85)	80(84)	68(84)	90(83)	58(84)
To improve the quality of how the research is reported	60(68)	61(69)	65(68)	58(72)	69(63)	54(78)
To proof the manuscript	26(30)	35(40)	39(41)	22(27)	38(35)	23(33)
To detect fraud	24(27)	32(36)	32(34)	24(30)	33(30)	23(33)
**How aware are you of the following types of peer review.**						
**Single blind review**						
I have never heard of this type of peer review	34(39)	19(22)	38(40)	16(20)	44(40)	10(14)
I have heard of this type, but I do not understand how it works	17(19)	21(24)	24(25)	14(17)	27(25)	12(17)
I have heard of this type and understand how it works	36(41)	46(52)	33(35)	48(59)	36(33)	46(67)
Not answered	1(1)	2(2)	0	3(4)	2(2)	1(1)
**Double blind peer review**						
I have never heard of this type of peer review	29(33)	20(23)	36(38)	14(17)	42(39)	8(12)
I have heard of this type, but I do not understand how it works	20(23)	19(22)	23(24)	16(20)	28(26)	12(17)
I have heard of this type and understand how it works	38(43)	47(53)	36(38)	48(59)	37(34)	48(70)
Not answered	1(1)	2(2)	0	3(4)	2(2)	1(1)
**Open peer review**						
I have never heard of this type of peer review	27(31)	15(17)	29(31)	12(15)	34(31)	8(12)
I have heard of this type, but I do not understand how it works	27(31)	31(35)	34(36)	24(30)	41(38)	18(26)
I have heard of this type and understand how it works	33(38)	40(45)	32(34)	42(52)	32(29)	42(61)
Not answered	1(1)	2(2)	0	3(4)	2(2)	1(1)
**Collaborative peer reivew**						
I have never heard of this type of peer review	37(42)	36(41)	40(42)	32(40)	49(45)	24(35)
I have heard of this type, but I do not understand how it works	38(43)	38(43)	44(46)	33(41)	48(44)	29(42)
I have heard of this type and understand how it works	11(13)	12(14)	11(12)	12(15)	10(9)	14(20)
Not answered	2(2)	2(2)	1(1)	4(5)	2(2)	2(3)
**Post publication peer review**						
I have never heard of this type of peer review	45(51)	40(45)	45(47)	41(51)	57(52)	29(42)
I have heard of this type, but I do not understand how it works	25(28)	31(32)	32(34)	23(28)	32(29)	24(35)
I have heard of this type and understand how it works	16(18)	14(16)	18(19)	12(15)	17(16)	14(20)
Not answered	2(2)	3(3)	0	5(6)	3(3)	2(3)
**Decoupled peer review**						
I have never heard of this type of peer review	76(86)	68(77)	76(80)	68(84)	91(83)	55(80)
I have heard of this type, but I do not understand how it works	11(13)	13(15)	15(16)	9(11)	13(12)	11(16)
I have heard of this type and understand how it works	0	5(6)	4(4)	1(1)	3(3)	2(3)
Not answered	1(1)	2(2)	0	3(4)	2(2)	1(1)
**Please rate the following journals for trustworthiness of peer review.**						
**BMJ**						
I have never heard of this journal	0	1(1)	1(1)	0	0	1()1
I do not know anything about peer review in this journal	9(10)	7(8)	4(4)	11(14)	11(10)	5(7)
I neither trust nor mistrust peer review in this journal	12(14)	12(14)	14(15)	10(12)	14(13)	11(16)
I have some concerns about peer review in this journal	8(9)	7(8)	7(7)	9(11)	7(6)	9(13)
I trust peer review in this journal	56(64)	59(67)	68(72)	47(58)	74(68)	41(59)
Not answered	3(3)	2(2)	1(1)	4(5)	3(3)	2(3)
**BMC Medicine**						
I have never heard of this journal	30(34)	16(18)	34(36)	13(16)	36(33)	11(16)
I do not know anything about peer review in this journal	26(30)	24(27)	24(25)	25(31)	36(33)	15(22)
I neither trust nor mistrust peer review in this journal	14(16)	21(24)	18(19)	17(21)	17(16)	18(26)
I have some concerns about peer review in this journal	2(2)	3(3)	1(1)	4(5)	1(1)	4(6)
I trust peer review in this journal	11(13)	20(23)	14(15)	17(21)	13(12)	18(26)
Not answered	5(6)	4(4)	4(4)	5(6)	6(6)	3(4)
**The Lancet**						
I have never heard of this journal	0	1(1)	1(1)	0	0	1(1)
I do not know anything about peer review in this journal	9(10)	5(6)	4(4)	9(11)	10(9)	4(6)
I neither trust nor mistrust peer review in this journal	9(10)	15(17)	12(13)	12(15)	12(11)	12(17)
I have some concerns about peer review in this journal	1(1)	4(5)	2(2)	3(4)	3(3)	2(3)
I trust peer review in this journal	67(76)	60(68)	74(78)	54(67)	81(74)	48(70)
Not answered	2(2)	3(3)	2(2)	3(4)	3(3)	2(3)
**PLOS Medicine**						
I have never heard of this journal	25(28)	13(15)	27(28)	12(15)	31(28)	8(12)
I do not know anything about peer review in this journal	22(25)	18(20)	19(20)	20(25)	29(27)	12(17)
I neither trust nor mistrust peer review in this journal	18(20)	23(26)	22(23)	19(23)	24(22)	17(25)
I have some concerns about peer review in this journal	1()1	5(6)	3(3)	3(4)	3(3)	3(4)
I trust peer review in this journal	17(19)	24(27)	19(20)	22(27)	16(15)	25(36)
Not answered	5(6)	5(6)	5(5)	5(6)	6(6)	4(6)
**NEJM**						
I have never heard of this journal	0	1(1)	1(1)	0	0	1(1)
I do not know anything about peer review in this journal	9(10)	6(7)	6(6)	8(10)	13(12)	2(3)
I neither trust nor mistrust peer review in this journal	5(6)	13(15)	11(11)	7(9)	10(9)	9(13)
I have some concerns about peer review in this journal	2(2)	0	0	2(2)	2(2)	0
I trust peer review in this journal	70(80)	66(75)	76(80)	61(75)	82(75)	55(80)
Not answered	2(2)	2(2)	1(1)	3(4)	2(2)	2(3)
**F1000 Research**						
I have never heard of this journal	57(65)	50(57)	62(65)	46(57)	70(64)	39(57)
I do not know anything about peer review in this journal	18(20)	12(14)	13(14)	16(20)	18(17)	12(17)
I neither trust nor mistrust peer review in this journal	7(8)	13(15)	11(12)	9(11)	11(10)	9(13)
I have some concerns about peer review in this journal	0	6(7)	2(2)	4(5)	2(2)	4(6)
I trust peer review in this journal	1(1)	3(3)	3(3)	1(1)	2(2)	2(3)
Not answered	5(6)	4(5)	4(4)	5(6)	6(6)	3(4)
**The key journal in your field**						
I have never heard of this journal	8(9)	11(13)	7(7)	11(14)	14(13)	5(7)
I do not know anything about peer review in this journal	3(3)	4(5)	4(4)	3(4)	7(6)	0
I neither trust nor mistrust peer review in this journal	12(14)	13(15)	13(14)	12(15)	13(12)	13(19)
I have some concerns about peer review in this journal	18(20)	21(24)	22(23)	17(21)	20(18)	19(28)
I trust peer review in this journal	38(43)	32(36)	40(42)	31(38)	45(41)	26(38)

Not answered	9(10)	9(10)	9(9)	7(9)	10(9)	6(9)
**How far do you trust each type of peer review in comparison to single blind peer review?**						
**Double blind peer review**						
No difference in trust either way	18(20)	18(20)	15(16)	20(25)	19(17)	17(25)
I trust this type of peer review less than single blind	3(3)	3(3)	3(3)	4(5)	4(4)	3(4)
I trust this type of peer reviewe more than single blind	64(73)	62(70)	73(77)	53(65)	79(72)	48(70)
Not answered	3(3)	5(6)	4(4)	4(5)	7(6)	1(1)
**Open peer review**						
No difference in trust either way	27(31)	29(33)	27(28)	28(35)	33(30)	23(33)
I trust this type of peer review less than single blind	39(44)	35(40)	43(45)	32(40)	50(46)	25(36)
I trust this type of peer reviewe more than single blind	20(23)	18(20)	21(22)	17(21)	19(17)	19(28)
Not answered	2(2)	6(7)	4(4)	4(5)	7(6)	2(3)
**Collaborative peer review**						
No difference in trust either way	33(38)	42(48)	39(41)	35(43)	45(41)	31(45)
I trust this type of peer review less than single blind	17(19)	10(11)	13(14)	14(17)	13(12)	14(20)
I trust this type of peer reviewe more than single blind	33(38)	28(32)	36(38)	26(32)	40(37)	22(32)
Not answered	5(6)	8(7)	7(7)	6(7)	11(10)	2(3)
**Post-publication peer review**						
No difference in trust either way	25(28)	25(28)	28(29)	22(27)	35(32)	16(23)
I trust this type of peer review less than single blind	44(50)	45(51)	45(47)	44(54)	50(46)	39(57)
I trust this type of peer reviewe more than single blind	12(14)	11(13)	14(15)	9(11)	11(10)	12(17)
Not answered	7(8)	7(8)	8(8)	6(7)	13(12)	2(2)
**Decoupled peer review**						
No difference in trust either way	41(47)	42(48)	42(44)	40(49)	48(44)	36(52)
I trust this type of peer review less than single blind	20(23)	21(24)	22(23)	19(23)	22(20)	19(28)
I trust this type of peer reviewe more than single blind	20(23)	18(20)	23(24)	16(20)	27(25)	12(17)
Not answered	7(8)	7(8)	8(8)	6(7)	12(11)	2(3)
**Is it important to your clinical decision making whether an article has been peer reviewed?**						
Yes	71(81)	80(91)	78(82)	72(89)	85(78)	67(97)
No	16(18)	8(9)	16(17)	9(11)	24(22)	1(1)
Not answered	1(1)	0	1(1)	0	0	1(1)
**How often do you look up information in scientific journals to aid your clinical decision making?**						
At least once a week	35(40)	39(44)	34(36)	39(48)	45(41)	29(42)
At least once a month	31(35)	36(41)	35(37)	32(40)	36(33)	31(45)
At least once every 3 months	10(11)	9()10	13(14)	6(7)	14(13)	6(9)
At least once every 6 months	7(8)	2(20	6(6)	3(4)	8(7)	1(1)
At least once a year	3(3)	0	3(3)	1(1)	3(3)	1(1)
Less than once a year	1(1)	2(2)	3(3)	0	3(3)	0
Not answered	1(1)	0	1(1)	0	0	1(1)
**When looking at an article to inform a clinical decision do you**						
Never consider peer review	21(24)	21(24)	29(31)	13(16)	36(33)	8(12)
Only consider articles published in journals where I trust peer review	59(67)	58(66)	57(60)	60(74)	62(57)	55(80)
Only trust articles where I can see peer review reports.	0	3(3)	1(1)	2(2)	1(1)	2(3)
Not answered	8(9)	6(7)	8(8)	6(7)	0	4(6)
**When making a clinical decision, do you ever consider the type of peer review beyond whether an article has been peer reviewed?**						
Yes	14(16)	8(9)	6(6)	16(20)	11(10)	11(16)
No	71(81)	79(90)	87(92)	63(78)	95(87)	57(83)
Not answered	3(3)	1(1)	2(2)	2(2)	3(3)	1(1)
